# Fasting glucose improvement following a short-term, culturally adapted lifestyle intervention in Latino adults at risk for type 2 diabetes mellitus: a quasi-experimental study

**DOI:** 10.1186/s40795-025-01155-6

**Published:** 2025-09-02

**Authors:** Rosario Suárez, Ruth Guillén, Nicolás Rodríguez, Celina Andrade, Andri Matos, Estefania Bautista-Valarezo

**Affiliations:** 1 School of Medicine, Universidad Técnica Particular de Loja, Loja, Ecuador; 2 School of Allied Health, Eastwick College, Ramsey, NJ USA

**Keywords:** Type 2 diabetes mellitus, Fasting glucose, Lifestyle intervention, FINDRISC, Latin america, DPP

## Abstract

**Background:**

Type 2 diabetes mellitus (T2D) is an increasingly pressing public health concern in Latin America. Lifestyle modification strategies, such as the Diabetes Prevention Program (DPP) have demonstrated significant benefits in reducing diabetes risk. However, limited evidence exists regarding their effectiveness in Latino communities, particularly when implemented in culturally tailored formats.

**Methods:**

This quasi-experimental study was conducted in Loja, Ecuador, from November 2023 to February 2024. A total of 126 adults aged 18 to 75 years with a FINDRISC score of 12 or higher participated in a 10-session, culturally adapted lifestyle intervention based on the DPP model. The intervention addressed motivation, physical activity, and nutrition. Anthropometric measures, body composition, lipid profiles (total cholesterol, HDL-c, LDL-c, and triglycerides), and fasting glucose levels were assessed before and after the intervention.

**Results:**

Post-intervention results revealed a statistically significant reduction in fasting glucose (from 107.33 ± 20.09 to 104.80 ± 15.65 mg/dL; *p* = 0.030), while lipid parameters remained unchanged. Both sexes experienced reductions in weight, with women showing greater improvements in systolic blood pressure, body mass index, waist-to-hip ratio, and body fat mass (*p* < 0.05).

**Conclusions:**

These findings suggest that short-term, culturally adapted lifestyle interventions may offer a feasible and effective strategy to improve glycemic outcomes among Latino adults at risk for T2D in community settings.

**Supplementary Information:**

The online version contains supplementary material available at 10.1186/s40795-025-01155-6.

## Introduction

Type 2 diabetes mellitus (T2D) is a chronic, nontransmissible, multifactorial disease caused by both modifiable and nonmodifiable factors. These factors often act synergistically to alter glucose regulation, posing serious health risks and necessitating timely preventive interventions [1, 2]. Furthermore, T2D represents a major global public health challenge, with an estimated 589 million adults aged 20 to 79 currently living with diabetes, a number projected to increase to 853 million by 2050. In the South and Central America region, the International Diabetes Federation (IDF) reports a prevalence of 10% [3]. Alarmingly, over 252 million individuals are unaware of their condition, heightening their risk of severe complications and premature mortality [3]. In Ecuador, T2D ranks among the top five causes of overall mortality, with a prevalence of 5.55% among adults aged 20 to 79 years [4].

Impaired fasting glucose (IFG), defined as fasting plasma glucose levels between 100 and 125 mg/dL, has been identified as a critical early indicator of T2DM. Longitudinal evidence from the Multi-Ethnic Study of Atherosclerosis (MESA) demonstrated that individuals with IFG had a significantly elevated risk of progressing to T2DM, with a hazard ratio exceeding 10 after adjustment for demographic and metabolic factors [5]. Recent clinical trials have shown that reducing fasting glucose through structured lifestyle interventions can meaningfully lower the risk of diabetes onset. For example, in the Norfolk Diabetes Prevention Study, participants receiving a pragmatic, group-based lifestyle program experienced a 43% reduction in T2DM incidence compared to controls [6]. These findings underscore the importance of early identification and targeted intervention in individuals with IFG to prevent disease progression and reduce associated cardiometabolic risk [7]. Individuals with T2D commonly exhibit atherogenic dyslipidemia, characterized by reduced high-density lipoprotein cholesterol (HDL-c) and elevated levels of triglycerides and low-density lipoprotein cholesterol (LDL-C) [1]. While normal HDL-c concentrations confer protective effects against insulin resistance, hypertriglyceridemia represents an independent risk factor for cardiovascular complications in patients with T2D, thereby contributing to increased cardiovascular mortality [8].

Given the association between T2D and atherosclerotic processes, strengthening primary prevention strategies for individuals at risk of developing T2D is imperative. Lifestyle modification programs have demonstrated both efficacy and cost-effectiveness, establishing them as viable public health interventions to improve population health outcomes [9].

The Diabetes Prevention Program (DPP) is a landmark randomized clinical trial that evaluated the efficacy of intensive lifestyle interventions in preventing T2D among individuals with impaired glucose tolerance [10]. The intervention demonstrated significant reductions in blood glucose levels, delayed T2D onset, and improved lipid profiles. Long-term follow-up through the Diabetes Prevention Program Outcomes Study (DPPOS) revealed sustained reductions in diabetes incidence over a median follow-up period of 21 years [11, 12]. However, limited evidence exists regarding the effectiveness of the DPP among Latino populations [13, 14]. The cultural adaptation of the DPP for the Ecuadorian population was grounded in social cognitive theory, which explains human behavior as a dynamic interaction between personal factors, environmental influences, and behavior [15, 16]. This theoretical framework was particularly relevant to our intervention, as it acknowledges how cultural context shapes health behaviors through observational learning, self-efficacy beliefs, and outcome expectations. Recent implementations of diabetes prevention programs have demonstrated that theory-based interventions promote more sustainable behavior change, with a clear articulation of how specific program components target theoretical constructs [17, 18]. A recent systematic review and meta-analysis in the Horn of Africa found that fewer than half of patients with type 2 diabetes adhere to recommended dietary practices, highlighting persistent barriers and the need for culturally and con- textually adapted educational interventions [19]. Our adaptation process addressed surface structures by aligning program materials with observable characteristics of the target population, and deep structures by integrating cultural values and social contexts that influence health behaviors among Ecuadorians.

Despite the demonstrated efficacy of the Diabetes Prevention Program (DPP) across diverse populations, a significant research gap persists regarding culturally adapted diabetes prevention interventions specifically designed for Latino communities. Although Latinos constitute one of the fastest-growing demographic groups affected by T2D globally, they remain underrepresented in prevention research [20, 21]. The limited number of studies that have included Latino participants suggest that cultural factors substantially influence both intervention engagement and clinical outcomes, often yielding better participation and effectiveness compared with non- adapted programs [22, 23]. However, evidence from South American populations remains particularly scarce, as most studies have focused on Mexican American or Caribbean Latino groups, whose cultural contexts differ from those of Ecuadorian populations [24, 25].

This study employed a quasi-experimental design to evaluate the effectiveness of the intervention—an approach well suited to community-based prevention research, where randomization may not be feasible or ethical. This methodological choice aligns with established epidemiological practices that balance internal validity with real-world implementation conditions [21, 26]. Our design included pre- and post- intervention measurements of key biomarkers and risk assessment tools to quantify program effects while accounting for the natural context in which the intervention was delivered. The quasi-experimental approach enabled implementation within existing community structures, enhancing the potential for future scalability while maintaining sufficient methodological rigor to draw meaningful conclusions about effectiveness.

## Methods

### Study design and participants

This prospective, quasi-experimental study was conducted in Loja, Ecuador, between November 2023 and February 2024. A total of 1,388 workers from various private and public institutions affiliated with the Ecuadorian Institute of Social Security (IESS) Health Center were initially screened for eligibility. Of these, 499 individuals met the inclusion criteria: adults aged 18 to 75 years with a T2D risk score of ≥ 12 points based on the Finnish Diabetes Risk Score (FINDRISC), who voluntarily agreed to participate by signing an informed consent form. Exclusion criteria included a prior diagnosis of diabetes, cognitive impairment, and pregnancy.

**Fig. 1:**
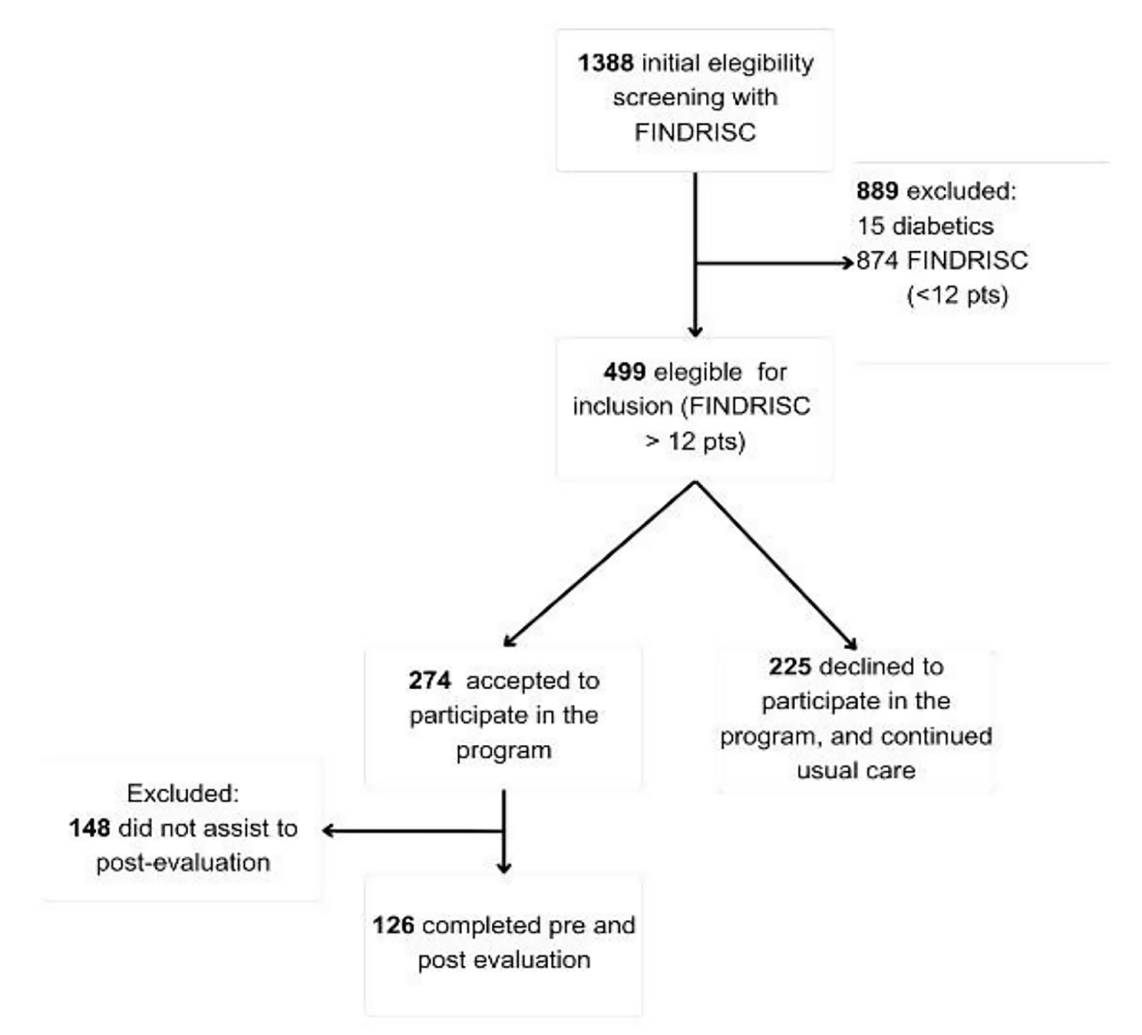
Flow chart of participants’ selection process

Among the eligible individuals, 274 consented to participate. However, owing to incomplete evaluations, the final analytical sample comprised 126 participants. Figure 1 outlines the participant selection process.

Baseline and postintervention assessments included fasting glucose levels and lipid profile components—total cholesterol, triglycerides, high-density lipoprotein cholesterol (HDL-c), and low-density lipoprotein cholesterol (LDL-c). The risk of T2D was measured using the FINDRISC score.

### Data collection and intervention design

Sociodemographic information, including sex, age, marital status, educational level, tobacco and alcohol use, was collected using a structured questionnaire specifically developed for this study (Supplementary Material 1). Physical activity level was assessed using the International Physical Activity Questionnaire (IPAQ) [27, 28]. Body composition parameters such as total fat mass, body fat percentage, skeletal muscle mass, weight, and body mass index (BMI) were measured using an InBody120 bioimpedance analyzer. Waist circumference was assessed with a Seca201 measuring tape (range: 0–205 cm; precision: ±0.9%). T2D risk was assessed using the Finnish Diabetes Risk Score (FINDRISC), which includes nine variables: age, sex, use of antihypertensive medication, history of hyper- glycemia, daily consumption of fruits and vegetables, daily physical activity of at least 30 min, family history of T2D (first- or second-degree relatives), BMI, and waist circumference. The total score ranges from 0 to 26 points; scores ≥ 12 are considered indicative of high T2D risk, according to the Ecuadorian Ministry of Public Health Diabetes Mellitus guidelines.

Fasting capillary blood samples were collected to measure glucose and lipid profile components, including total cholesterol (TC), triglycerides (TG), HDL cholesterol (HDL-c), and LDL cholesterol (LDL-c). Glucose concentrations were measured using an Accu-Chek Instant^®^ glucometer compliant with ISO 15197:2013/EN ISO 15197:2015 standards [29]. Lipid parameters were measured using the LipidoCare^®^ Standard Biosensor system with its corresponding test strips. This device is certified by the Cholesterol Reference Method Laboratory Network (CRMLN) and demonstrates a variation of approximately ± 5–10% compared with conventional laboratory methods; its reliability has been validated in previous studies [30, 31]. All procedures were conducted in the morning after a minimum eight-hour overnight fast. Participants were instructed to have a light dinner the evening before and to rest adequately prior to sample collection. Biohazard precautions were followed throughout.

Following baseline assessments, participants completed a culturally adapted lifestyle intervention based on the Diabetes Prevention Program (DPP), titled Trans- form Your Life with Daily Changes. The original 22-session curriculum was condensed into 10 structured sessions, grouped into three thematic domains: motivation (M), physical activity (PA), and nutrition (Nt). This adaptation was designed to accommodate the full-time work schedules of participants employed in public and private institutions, to maximize feasibility and resource efficiency. Cultural tailoring included the integration of region-specific foods and locally preferred physical activities (e.g., dancing and recreational running). The intervention focused on three main pillars: (a) regular and culturally appropriate physical activity; (b) adherence to a healthy diet; and (c) motivational strategies to enhance long-term adherence. Participants were provided with practical guidance on healthy food choices, portion sizes, calorie control, aerobic and resistance exercise routines, and cognitive strategies to overcome negative thought patterns and support sustainable lifestyle change.

**Fig. 2:**
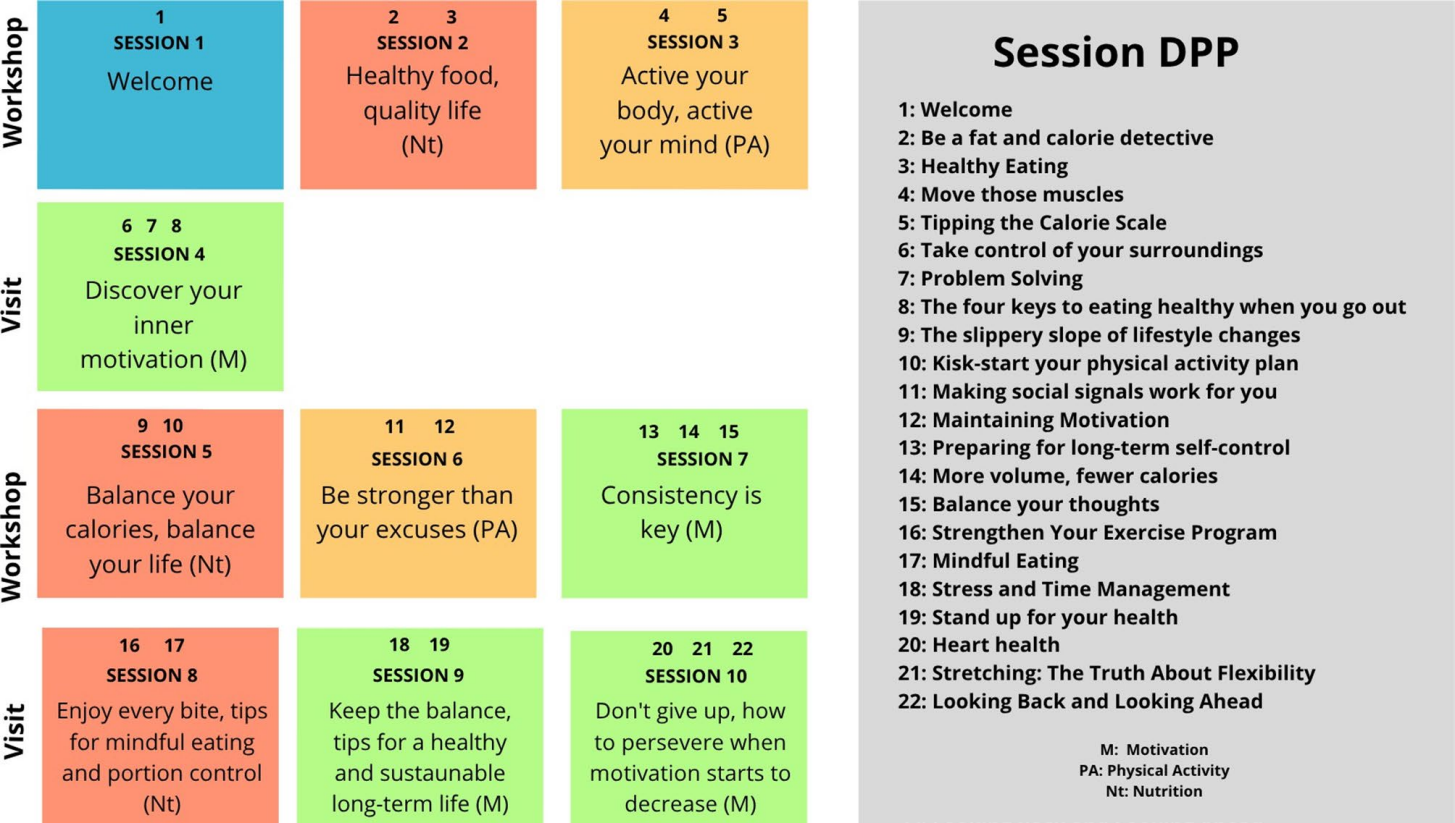
DPP principles and their adaptation to the “Transform your life with daily changes” program

At the end of the intervention, all baseline assessments—including FINDRISC score, anthropometric and body composition measures, fasting glucose, and lipid profile—were repeated using the same standardized protocols. Figure 2 presents the original DPP principles and their restructuring into the 10-session format used in this study.

Additionally, an exit survey was conducted to offer a systematic assessment of participants’ opinions on several aspects, including perceived information quality, support, understanding of the content, educational resources, motivating factors, and suggestions for program improvement. The development and use of such a survey ensures that both objective outcomes and subjective experiences are systematically documented, which is consistent with recommendations for participant-centered assessment in lifestyle interventions [32, 33]. Moreover, a stratified analysis of session attendance by gender was conducted to examine adherence patterns and potential disparities.

### Ethical considerations

Ethical approval for this study was obtained from the Institutional Ethics Committee of the Hospital San Francisco, Quito, Ecuador (Institutional Review Board approval code 031). Written informed consent was obtained from all participants. Data confidentiality was safeguarded through anonymization procedures. This study was carried out taking into consideration the ethical principles of the Declaration of Helsinki.

### Statistical analysis

Statistical analyses were performed using IBM SPSS Statistics version 26 (IBM Corp., Armonk, NY, USA). Categorical variables were summarized as frequencies and percentages. Continuous variables were reported as means with standard deviations (SD) or medians with interquartile ranges (IQR), depending on distribution.

Normality of continuous variables was assessed using the Kolmogorov–Smirnov test and visual inspection of histograms. For normally distributed variables with homogeneous variance, pre- and post-intervention comparisons were conducted using the paired Student’s t -test. For non-normally distributed variables, the Wilcoxon signed-rank test was applied. A two-sided p-value < 0.05 was considered statistically significant.

## Results

A total of 126 participants (40 men and 86 women) completed the intervention along with both pre- and post-assessments. Sociodemographic characteristics of the sample are presented in Table 1. The mean age was 47 years for men and 46 years for women. A significantly higher proportion of women held university or postgraduate degrees compared to men (*p* = 0.04). Regarding marital status, married individuals predominated among men (77.5%), while women showed a more heterogeneous distribution (*p* = 0.004). No significant sex differences were observed in alcohol or tobacco use. However, physical activity levels were significantly lower in women compared to men (*p* < 0.001), although sedentary time was similar between sexes.

**Table 1:** Sociodemographic characteristics of participants at baseline

Variable	Male (*n* = 40)	Female (*n* = 86)	*p*-value
Age, years (M ± SD)	47 (9)	46 (9)	0.123
Education, n (%)			0.0401^1*^
Lower than high school	2 (5)	1 (1.2)	
High school	10 (25)	8 (9.3)	
College	19 (47.5)	45 (52.3)	
Graduate	9 (22.5)	32 (37.2)	
Marital Status, n (%)			0.0042^2*^
Single	5 (12.5)	21 (24.4)	
Married	31 (77.5)	42 (48.8)	
Divorced	1 (2.5)	16 (18.6)	
Widowed	0 (0)	5 (5.8)	
De facto union	3 (7.5)	2 (2.3)	
Current smokers, n (%)	8 (20)	7 (8.1)	0.056
Alcohol use (past 30 days), n (%)	21 (52.5)	40 (46.5)	0.531
Weekly PA level, n (%)			< 0.0013^3*^
Low	16 (40)	61 (70.9)	
Moderate	10 (25)	20 (23.3)	
High	14 (35)	5 (5.8)	
Sedentary time, hours/day (M ± SD)	5.06 (3.20)	4.72 (3.21)	0.582

^1^Chi-square test for educational level; ^2^Chi-square test for marital status; ^3^Chi-square test for physical activity level; *p-value < 0.05. M: mean; SD: standard deviation

Tables 2 and 3 present the pre- and post-intervention anthropometric and body composition measurements for men and women, respectively. In men, a statistically significant reduction in body weight was observed (83.3 ± 11.15 kg to 81.99 ± 11.55 kg; *p* < 0.001), with a mean weight loss of 1.3 kg (*p* = 0.010). Other variables showed favorable trends, including reductions in waist circumference and body fat mass, but these trends did not reach statistical significance.

In women, several parameters significantly improved: BMI (28.83 ± 3.85 to 27.31 ± 3.88 kg/m2; *p* < 0.001), waist–hip ratio (0.94 ± 0.05 to 0.93 ± 0.05; *p* < 0.001), body fat mass (28.57 ± 7.31 to 27.65 ± 7.16 kg; *p* = 0.002), systolic blood pressure (121.84 ± 13.72 to 117.14 ± 14.18 mmHg; *p* < 0.001), and weight (68.64 ± 10.39 to 67.42 ± 10.20 kg; *p* = 0.001). The increase in diastolic blood pressure was not considered clinically relevant, as it primarily reflected a shift from subnormal to normal values.

**Table 2:** Changes in anthropometric and cardiovascular parameters among men before and after the intervention

Parameter	Pre-intervention (Mean ± SD)	Post-intervention (Mean ± SD)	*p*-value
BMI (kg/m2)	29.63 (3.58)	29.36 (3.69)	0.183
Waist circumference (cm)	103.31 (14.36)	99.74 (9.95)	0.072
WHR	0.95 (0.04)	0.94 (0.04)	0.098
Muscle mass (kg)	31.41 (4.49)	31.39 (4.26)	0.971
Body fat percentage (%)	32.34 (6.02)	32.02 (6.45)	0.442
Body fat mass (kg)	27.45 (7.39)	26.63 (7.63)	0.101
Visceral fat level	11.90 (3.81)	12.01 (5.20)	0.831
SBP (mmHg)	131.60 (15.45)	127.58 (12.35)	0.075
DBP (mmHg)	81.80 (9.95)	81.53 (9.05)	0.859
Weight (kg)	83.30 (11.15)	81.99 (11.55)	< 0.001*
Weight loss (kg)	—	1.30 (3.05)	0.010*

*p-value < 0.05. BMI: body mass index; WHR: waist-to-hip ratio; SBP: systolic blood pressure; DBP: diastolic blood pressure

**Table 3:** Changes in anthropometric and cardiovascular parameters among women before and after the intervention

Parameter	Pre-intervention (Mean ± SD)	Post-intervention (Mean ± SD)	*p*-value
BMI (kg/m2)	28.83 (3.85)	27.31 (3.88)	< 0.001*
Waist circumference (cm)	92.61 (8.95)	91.63 (8.89)	0.112
WHR	0.94 (0.05)	0.93 (0.05)	< 0.001*
Muscle mass (kg)	22.09 (3.20)	21.74 (3.15)	0.186
Body fat percentage (%)	41.01 (5.45)	40.49 (5.68)	0.122
Body fat mass (kg)	28.57 (7.31)	27.65 (7.16)	0.002*
Visceral fat level	13.80 (3.82)	13.44 (4.16)	0.100
SBP (mmHg)	121.84 (13.72)	117.14 (14.18)	< 0.001*
DBP (mmHg)	72.91 (10.27)	76.73 (9.97)	< 0.001*
Weight (kg)	68.64 (10.39)	67.42 (10.20)	0.001*
Weight loss (kg)	—	1.22 (3.37)	—

*p-value < 0.05. BMI: body mass index; WHR: waist-to-hip ratio; SBP: systolic blood pressure; DBP: diastolic blood pressure

Table 4 presents the biochemical outcomes before and after the intervention. A statistically significant reduction in fasting glucose levels was observed (*p* = 0.030). Although triglyceride levels decreased modestly and HDL-c levels increased slightly, these changes were not statistically significant (*p* > 0.05). Similarly, total cholesterol and LDL-c levels remained unchanged following the intervention.

**Table 4:** Participants’ glucose and lipid profiles before and after the intervention

Parameters	Preintervention	Postintervention	*p*-value
Fasting glucose (mg/dL)	107.33 (20.09)	104.80 (15.65)	0.030*
Total cholesterol (mg/dL)	191.10 (50.09)	195.18 (43.57)	0.476
Triglycerides (mg/dL)	199.37 (118.52)	190.08 (105.41)	0.533
HDL-c (mg/dL)	49.82 (9.59)	52.97 (11.27)	0.126
LDL-c (mg/dL)	100.85 (38.83)	103.81 (34.64)	0.577
*p-value < 0.05			

Additionally, we assessed the participants’ perspectives on the program with an exploratory survey of their opinions over seven topics that are presented in Table 5.

**Table 5:** Summary of the participants’ perspectives on the program, exit survey

		*n*	%
Quality of Information	very poor	0	0,0%
	poor	0	0,0%
	fair	0	0,0%
	good	11	8,8%
	excellent	114	91,2%
Understanding of Content	completely understood	123	98,4%
	partially understood	0	0%
	did not understand	2	1,6%
Level of Support	very poor	0	0,0%
	poor	0	0,0%
	fair	0	0,0%
	good	19	15,2%
	excellent	106	84,8%
Educational material	very poor	0	0,0%
	poor	0	0,0%
	fair	0	0,0%
	good	34	27,2%
	excellent	91	72,8%
Reported Positive Changes	yes	121	97,6%
	no	0	0,0%
	not sure	3	2,4%
Sources of Motivation When Discouraged	testimonials	4	3,2%
	provided information and videos	54	43,2%
	Improved eating habits	24	19,2%
	health gains	28	22,4%
	maintaining healthy weight	6	4,8%
	none	9	7,2%
Suggestions for Program Improvement	greater emphasis on physical activity	5	4,0%
	more continuos follow-up	26	20,8%
	greater emphasis on nutrition	10	8,0%
	longer program duration and better organization	21	16,8%
	Increased participant interaction	8	6,4%
	Include more participants	8	6,4%
	no suggestions	47	37,6%

Table 5 compiles the views of 125 participants who completed the exit survey. Overall, 91.2% of respondents rated the material as “excellent,” compared to 8.8% who rated it as “good.” 98.4% of respondents claimed to have “Completely Understood” the material. 84.8% of respondents gave program support an “Excellent” rating, while 15.2% gave it a “Good” grade. Similar ratings were given to the training materials: 27.2% said they were “Good,” and 72.8% said they were “Excellent.” 97.6% of respondents said the intervention had a positive effect on them. 43.2% of respondents cited the videos and information they were provided as the reason they kept going when they were feeling discouraged, 22.4% said that feeling that their health had improved, and 19.2% claimed that the consciousness that they were eating better helped them to keep moving forward. More continuous follow-up was the most often suggested improvement (20.8%), followed by requests for a longer program duration and improved organization (16.8%); however, 37.6% of respondents made no suggestions at all.

**Table 6:** Attendances to sessions

Variable	Male (*n* = 40)	Female (*n* = 86)
Sessions attended, n (%)		
8 to 10 attendances	7 (17.5)	15 (17.4)
4 to 7 attendances	19 (47.5)	53 (61.6)
1 to 3 attendances	14 (35)	18 (20.9)

**Fig. 3:**
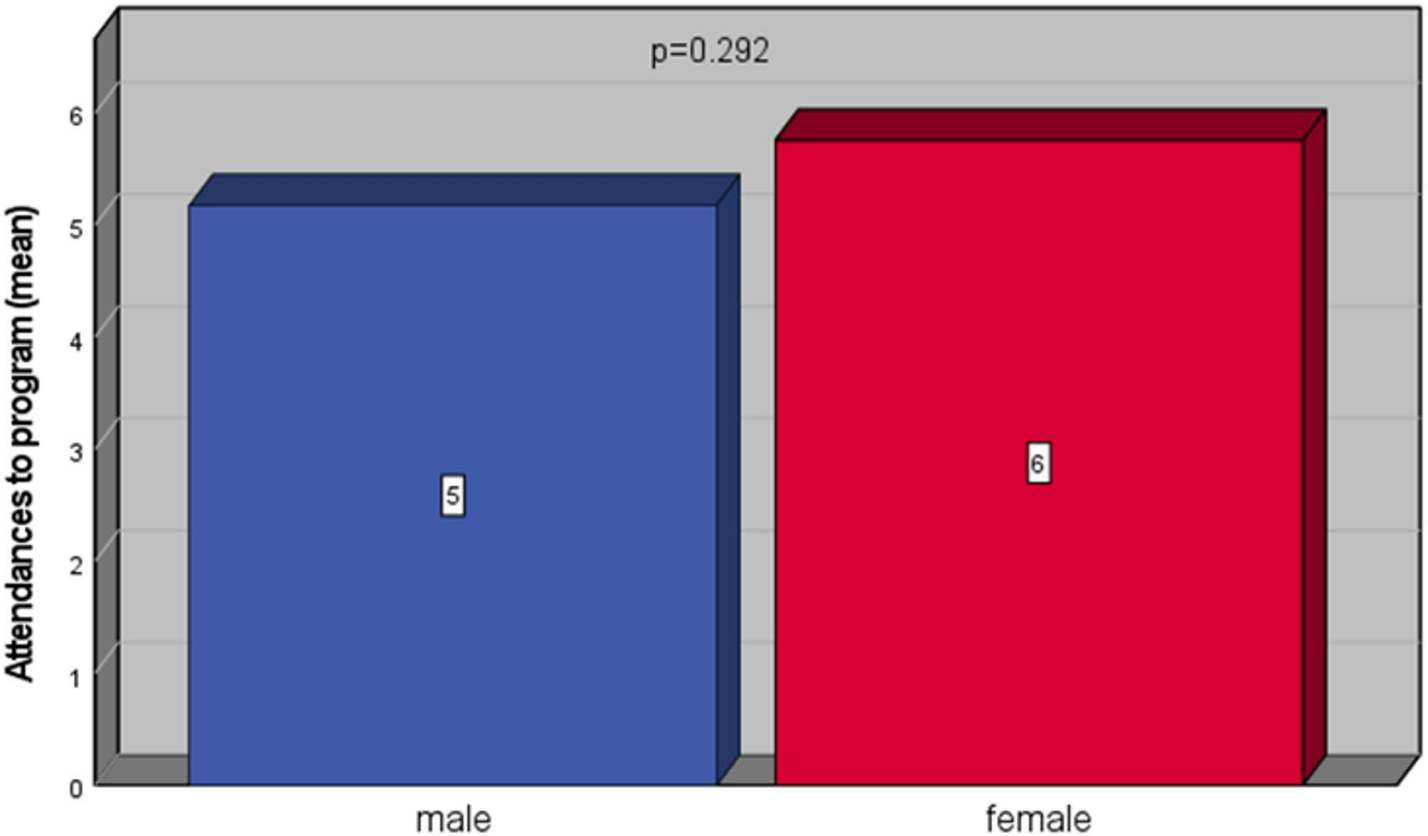
Average differences in program attendance between genders

Table 6 summarizes session attendance by gender. In both men and women, the largest proportion attended 4–7 sessions (47.5% of men vs. 61.6% of women). Meanwhile, 17.5% of men and 17.4% of women complete 8–10 sessions. The difference in overall attendance rates between genders did not reach statistical significance. Nevertheless, women exhibited a higher prevalence of moderate attendance (4–7 sessions) and a lower of minimal attendance (1–3 sessions), indicating a qualitative tendency toward greater adherence among female participants. Figure 3 illustrate the mean difference in sessions attendance between men and women.

## Discussion

The present study demonstrated that a 16-week, culturally adapted intervention based on the Diabetes Prevention Program (DPP) achieved a statistically significant reduction in fasting glucose (FG) levels among Ecuadorian adults at high risk for T2D, with women exhibiting particularly pronounced improvements in anthropometric and metabolic parameters. These findings align with established evidence that lifestyle interventions can modulate glycemic control in prediabetic populations while extending current knowledge by highlighting the feasibility of condensing traditional DPP formats without sacrificing clinical efficacy [6, 34, 35].

The observed mean fasting glucose reduction of 2.53 mg/dL (from 107.33 to 104.80 mg/dL; *p* = 0.030) is clinically relevant, given the linear association between fasting glucose and both diabetes and cardiovascular risk [36, 37]. Despite the intervention’s shorter duration compared to conventional DPP models, the results suggest that strategic cultural adaptations may enhance intervention efficiency in Latino populations [14, 38].

In Ecuador, a previous pilot study involving 33 adults with prediabetes implemented a 6-month DPP-based intervention and reported a mean HbA1c reduction of 0.84%, although fasting glucose was not assessed [24]. Our findings are consistent with prior systematic reviews and meta-analyses that have demonstrated similar reductions in fasting glucose across various lifestyle intervention programs [9, 39, 40].

Sex-specific outcomes revealed that women experienced significant reductions in BMI (− 1.52 kg/m2), body fat mass (− 0.92 kg), and systolic blood pressure (− 4.70 mmHg). These findings are consistent with results from the Lawrence Latino Diabetes Prevention Project, which attributed gender-based differences to greater engagement by women in dietary modifications and stronger reliance on social support networks [41]. This trend may be partly explained by cultural dynamics within Ecuadorian com- munities, where women often bear primary responsibility for household nutrition and face heightened societal pressure to adhere to body weight norms [42, 43].

In contrast, no significant changes were observed in lipid profile parameters. This finding diverges from several meta-analyses that reported improvements in lipid profiles following lifestyle interventions [44]. However, our results are consistent with evidence suggesting that such lipid profile changes typically emerge after longer intervention periods (e.g., ≥ 12 months) and in the context of more substantial weight loss [45, 46].

It is also important to consider participants’ baseline dietary patterns. For instance, low-fat diets are associated with reductions in LDL-c but may increase triglyceride levels, whereas low-carbohydrate diets tend to produce the opposite effect [47]. The findings of the present study may be partially explained by suboptimal fat intake regulation, despite potential reductions in carbohydrate and refined sugar consumption.

### Contextualizing results within Latino health disparities

Our findings contribute valuable data to the underexplored area of diabetes prevention among South American Latino populations, addressing a persistent research gap in which nearly 80% of Latino DPP studies have focused primarily on Mexican American or Caribbean subgroups [48]. The observed improvements in glycemic control, despite limited lipid changes, highlight the complex interplay between physiological outcomes and the depth of cultural adaptation [20].

Structural adaptations specific to Ecuador, such as limited time for healthy meal preparation and sedentary occupational patterns in formal employment sectors— may need to be further intensified to achieve broader metabolic improvements. Nevertheless, surface-level adaptations (e.g., using locally available foods and incorporating dance-based physical activity) likely contributed to improved participant retention [49].

The absence of a significant reduction in triglyceride levels (− 3.74 mg/dL; *p* = 0.696) contrasts with the DPPOS findings, which reported 20–30 mg/dL reductions following similar interventions [1, 12]. This discrepancy may reflect unique dietary patterns in Andean populations. Compared with our modified Mediterranean approach, traditional Ecuadorian diets—often rich in refined carbohydrates—may require more intensive carbohydrate restriction to achieve lipid improvements. Recent evidence supports this hypothesis, linking high-glycemic foods to blunted lipid responses during lifestyle interventions [50, 51].

On the other hand, when examining the overwhelmingly positive responses regarding participants ‘perceptions about the program, including the high percentages indicating complete understanding, excellent quality of information and materials, and positive perceived changes, these are consistent with recent research showing how important participant satisfaction and comprehension are to adherence and intervention outcomes [52, 53]. Strong support and high-quality learning resources are regularly associated with increased motivation, engagement, and program retention as well as a stronger desire to make significant behavioral changes. Furthermore, the usefulness of multimodal, tailored material for maintaining engagement is reinforced by the discovery of motivational factors when participants feel discouraged, particularly the effectiveness of videos, testimonials, and observable health benefits [32, 54].

Furthermore, gender-based analysis is consistent with existing research showing that overall session attendance does not differ significantly by gender, even though women frequently exhibit higher enrollment rates and moderate attendance in nutrition programs [55]. This pattern indicates that when programs are well-designed and fit participant requirements, both genders can adhere strongly. However, to improve retention, program planners should continue to pay attention to gender-based preferences [56, 57].

These findings emphasize the need of using systematic participant input and comprehensive attendance data in the design and evaluation of nutrition treatments, which is consistent with recent suggestions to standardize mixed-methods process assessment in intervention research. By considering participants’ viewpoints and engagement patterns, programs can be more effectively adjusted to use facilitators and address identified barriers, thereby expanding their reach and effectiveness [58].

### Limitations

The quasi-experimental design, while appropriate for real-world implementation, limits the ability to attribute observed changes exclusively to the intervention. A potential source of selection bias is the 27.6% attrition rate (148 of 274 participants excluded due to incomplete data), which may overrepresent individuals with higher motivation levels.

Although the biochemical measurements were validated against laboratory procedures, the use of capillary sampling introduces inherent variability (approximately ± 10%), potentially obscuring subtle changes in lipid parameters. Furthermore, the condensed format of the intervention (10 sessions versus the original 22 in the DPP) raises concerns regarding long-term sustainability. The DPP Outcomes Study (DPPOS) suggests that programs with 16 or more sessions are associated with superior 10-year reductions in diabetes incidence [12].

Nevertheless, recent meta-analyses and systematic reviews have demonstrated that short-term lifestyle interventions (≤ 6 months) can significantly improve cardiometabolic indicators—including body weight, glycemia, and insulin resistance—among adults with overweight, obesity, or metabolic syndrome [35, 59, 60]. Importantly, the high postintervention retention rate (126 of 126) observed in our study suggests that the cultural adaptation of the program likely mitigated the attrition commonly reported in abbreviated interventions.

While this study did not assess cardiac outcomes, prior evidence suggests that hyperglycemia, even before formal diabetes diagnosis, can induce subclinical cardiac changes. Cardiac MRI studies in newly diagnosed type 2 diabetes have revealed increased myocardial mass and concentric remodeling [61], while gestational diabetes has been linked to early ventricular dysfunction despite transient glycemic elevation [62]. Future research should examine whether glycemic improvements from short-term interventions correlate with reduced or reversible cardiac remodeling.

### Strengths and innovations

This study contributes to Latino health research through three key innovations:

Rigorous cultural adaptation: The intervention systematically incorporated components of social cognitive theory—such as self-efficacy building through “*grupos de apoyo”* and observational learning via meal preparation demonstrations, addressing both surface and deep cultural structures. This approach has been shown to double adherence rates in Latino-focused trials [63–65].

Sex-stratified analysis: By examining gender-specific outcomes, the study identified actionable targets for future interventions. Specifically, it highlighted the need to engage Ecuadorian men through workplace-based strategies and culturally congruent physical activities, such as neighborhood soccer leagues.

Biomarker transparency: Comprehensive reporting of null lipid outcomes counters publication bias that often affects Latino health literature and provides valuable data for future meta-analyses.

Furthermore, our findings align with the ecological validity model, which emphasizes adapting interventions to local contexts—for example, by incorporating workplace activity breaks or improving cafeteria offerings [66]. Digital health tools may also help overcome scheduling barriers while preserving cultural relevance [67–69].

## Conclusion

This quasi-experimental study provides preliminary evidence supporting the effectiveness of a culturally adapted, short-term lifestyle intervention—grounded in the Diabetes Prevention Program (DPP)—in reducing fasting glucose levels among Latino adults at high risk for T2D in Ecuador. The intervention led to statistically significant improvements in glycemic control and modest anthropometric changes, particularly among women, despite no significant alterations in lipid profiles. These findings suggest that culturally tailored, time-efficient programs can enhance participant engagement and retention, making them promising tools for diabetes prevention in underserved Latino communities.

This study offers novel insights through the systematic cultural adaptation of the DPP, incorporating social cognitive theory, locally relevant content and behaviors, and sex-stratified outcome analyses. Nonetheless, limitations include the absence of a control group, potential selection bias due to attrition, and a short follow-up period, which constrain causal inference and long-term generalizability.

Future research should incorporate randomized controlled designs, longer follow- up durations, and more intensive dietary modifications to confirm and extend these findings. Scaling culturally responsive interventions may help bridge diabetes prevention gaps in South American populations, contributing to more equitable and context-sensitive public health strategies.

## Supplementary Information

Below is the link to the electronic supplementary material.

Supplementary Material 1

## Data Availability

The datasets used and/or analyzed during the current study are available from the corresponding author upon reasonable request. Interested researchers may contact the corresponding author, Dr. Rosario Suárez, at [rsuarez2@utpl.edu.ec](mailto: rsuarez2@utpl.edu.ec).

## Abbreviations

IDF: International Diabetes Federation
T2D: Type 2 Diabetes
DPP: Diabetes Prevention Program
FG: Fasting glucose
HbA1c: Glycated hemoglobin
HDL-c: Cholesterol bound to high-density lipoproteins
LDL-c: Cholesterol bound to low-density lipoproteins
PTOG: Oral glucose tolerance test
T2DR: Type 2 diabetes risk
CR: Caloric restriction
TC: Total cholesterol
TG: Triglycerides
WHO: World Health Organization
