# Supplementary material for: Fasting glucose improvement following a short-term, culturally adapted lifestyle intervention in Latino adults at risk for type 2 diabetes mellitus: a quasi-experimental study
Source: BMC Nutr. 2025 Sep 2;11:171. doi: 10.1186/s40795-025-01155-6 (PMC12403463; doi:10.1186/s40795-025-01155-6)
Supplement: Supplementary file 1 — Supplementary Material 1 [file 40795_2025_1155_MOESM1_ESM.docx]

| **Demographic information** | | | **Answer** | **Code** |
| --- | --- | --- | --- | --- |
| 11 | AGE | How old are you? | Years________ |  |
| 12 | F_NACE | What is your **date of birth** ? | ___ ___ ______  Day Month Year  Don't know 77 |  |
| 13 | SEX | Sex | Male 1  Female 2 |  |
| 14 | N_INSTRUC | What is the highest **level of education you have achieved?** | None 0  Primary school completed 1  Secondary school completed 2  Completed university degree (degree) 3  Postgraduate completed 4 |  |
| 15 | EST_CIV | What is your **marital status** ? | Single 1  Married 2  Divorced 3  Widower 4  Free union 5 |  |

| **Behavioral/habit measurements: TOBACCO** | | | | **Answer** | **Code** |
| --- | --- | --- | --- | --- | --- |
| 18 | SMOKE | Do you **currently smoke any tobacco products** such as cigarettes, cigars, pipes, or vapes (electronic cigarettes)? | | Does not smoke 0  If you smoke 1  If the answer is No, skip to question 22 |  |
| 22 | EX_FUM | Have you ever been a smoker? | | No 0  Yes 1  If the answer is NO, skip to question 24 |  |
| **Behavioral/habit measurements: ALCOHOL** | | | **Answer** | | **Code** |
| 24 | CONSUME ALCOHOL | Have you **ever** consumed any alcoholic beverage such as beer, wine, brandy, or other? | No 0  Yes 1  If the answer is NO, skip to question 32 | |  |
| 27 | CONSUM_ALCOH_30 | Have you consumed an alcoholic beverage within the **last 30 days** ? | No 0  Yes 1  If the answer is NO, skip to question 32 | |  |

| **Behavioral/habit measurements: PHYSICAL ACTIVITY (IPAQ Questionnaire)**  We are interested in learning about the type of physical activity you do in your daily life. The questions refer to the last 7 days. Please fill in the blanks. | | | **Answer** | **Code** |
| --- | --- | --- | --- | --- |
| 32 | DAYS_ACT_FIS_INT | During the last 7 days, on how many days did you perform **Intense physical activities such as lifting heavy weights, digging, playing soccer, exercises like aerobics, running or riding a fast bike** ? | Number of days per week ____  Don't know 77  Without intense physical activity, skip to question 38 |  |
| 33 | TIEM_ACT_FIS_INT | Typically, **how much total time did you spend on vigorous** physical activity on one of those days? (write in multiples of 10 minutes) | Number of hours per day ____  Number of minutes per day ____  Don't know 77 |  |
| 34 | DAYS_ACT_FIS_MOD | During the past 7 days, on how many days did you do **moderate physical activity, such as carrying light weights (less than 20 kg) or cycling at a regular speed? Do not include walking.** | Number of days per week ____  Don't know 77  Without moderate physical activity, skip to question 36 |  |
| 35 | TIEM_ACT_FIS_MOD | Typically, **how much total time did you spend on moderate** physical activity on one of those days? | Number of hours per day ____  Number of minutes per day ____  Don't know 77 |  |
| 36 | DAYS_WAY | During the last 7 days, how many days **did you walk** for at least 10 minutes at a time? | Number of days per week ____  Don't know 77  If you did not walk, skip to question 42 |  |
| 37 | TIME_WAY | Typically, **how much time** in total did you spend **walking** on one of those days? | Number of hours per day _____  Number of minutes per day _____  Don't know 77 |  |
| 38 | SITTING_HOURS | During the last 7 days, how much time did you spend **sitting** during a workday? | Number of hours per day _____  Number of minutes per day _____  Don't know 77 |  |
